# Marked Hyperbilirubinemia Associated with Primary Myelofibrosis Responsive to Ruxolitinib

**DOI:** 10.1155/2022/9630996

**Published:** 2022-05-28

**Authors:** Sunny Sandhu, Hunza Chaudhry, Sharon Zhang, Devang Prajapati

**Affiliations:** ^1^Department of Internal Medicine, University of California, San Francisco- Fresno, Fresno, CA, USA; ^2^Department of Pathology, Community Medical Centers, Fresno, CA, USA; ^3^Department of Gastroenterology & Hepatology, University of California, San Francisco- Fresno, Fresno, CA, USA

## Abstract

Primary myelofibrosis (PMF) is a chronic myeloproliferative disorder seen in older adults which can present both with hepatosplenomegaly as well as mild nonspecific liver enzyme abnormalities. Mild elevations in bilirubin can occasionally be seen due to both intravascular hemolysis as well as extramedullary hematopoiesis. Marked hyperbilirubinemia as a presenting sign of PMF progression, however, has not been reported. We present the case of a patient with a remote diagnosis of PMF, who presented with marked hyperbilirubinemia with a notable response to ruxolitinib.

## 1. Introduction

Primary myelofibrosis (PMF) is a chronic myeloproliferative disorder most commonly seen in older adults which can commonly lead to liver and spleen involvement due to extramedullary hematopoiesis (EMH). Mild hyperbilirubinemia can also be seen due to increased red blood cell turnover. We describe an unusual case of cholestatic liver injury with marked hyperbilirubinemia in a patient with a remote diagnosis of PMF, who had a notable response to ruxolitinib. There are no other similar reported cases in the literature to the best of our knowledge.

## 2. Case Presentation

**Figure 1:**
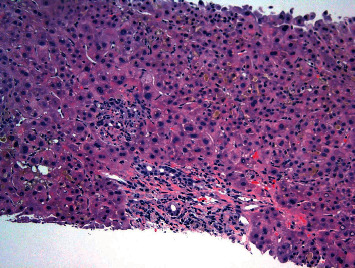
H&E stain (200×) showing marked canalicular cholestasis, portal and periportal fibrosis, bile duct injury, ductular reaction, and portal and lobular neutrophilic infiltrates, most suggestive of biliary obstruction.

**Figure 2:**
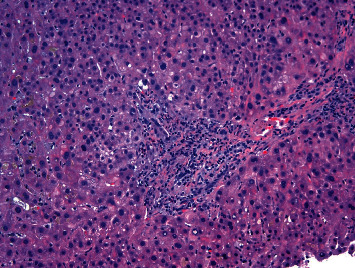
H&E stain (200×) showing marked canalicular cholestasis, portal and periportal fibrosis, bile duct injury, ductular reaction, and portal and lobular neutrophilic infiltrates, most suggestive of biliary obstruction.

**Figure 3:**
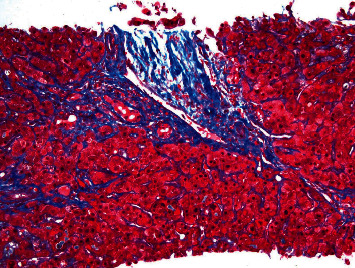
Trichrome stain (40×) showing portal and periportal fibrosis, as well as focal sinusoidal collagen deposition.

**Figure 4:**
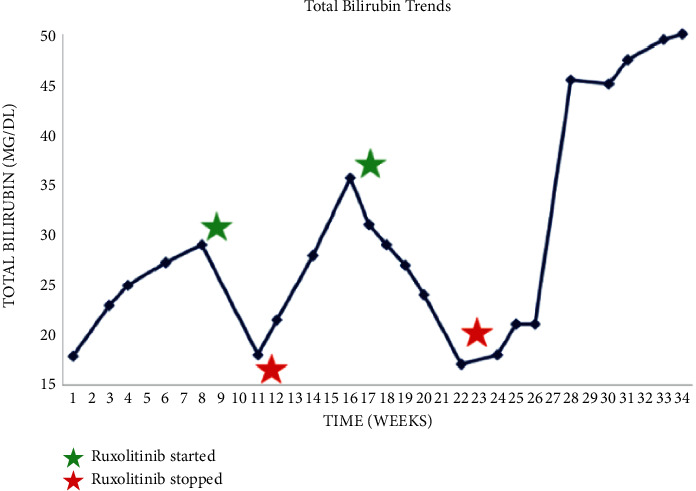
Total bilirubin trends with relation to initiation and discontinuation of ruxolitinib.

A 64-year-old male with a 10-year history of asymptomatic JAK-2-positive PMF under observation presented with progressive pruritus and jaundice for 3 days. Labs revealed ALT 49 IU/L, AST 35 IU/L, ALP 378 IU/L, total bilirubin (TB) 17 mg/dL, and direct bilirubin 10 mg/dL. LDH was 173 U/L, and the haptoglobin level was normal. Complete blood count with differential showed WBC 4.4 × 10^3^/uL (85% neutrophils, 8% lymphocytes, and 4% monocytes), hemoglobin 7.4 g/dL, and platelets 103 × 10^9^/L. Lipid panel showed total cholesterol 229 mg/dL, triglyceride 171 mg/dL, HDL 5 mg/dL, and LDL 193 mg/dL. Anti-mitochondrial antibody was negative with titer <1 : 20. Workup for metabolic, viral, autoimmune, alcoholic, and drug-induced liver disease otherwise was unrevealing. The patient denied the use of any drugs, including herbal supplements. CT scan showed hepatomegaly without biliary ductal dilatation, along with marked splenomegaly with a spleen size of 27 cm. MRCP failed to demonstrate findings of an obstructive etiology. Therefore, EUS and ERCP were performed which were also unremarkable. Given worsening hyperbilirubinemia, a liver biopsy was subsequently pursued which was suggestive of a nonspecific cholestatic liver injury (Figures 1–3). TB continued to increase to 25 mg/dL, and a repeat ERCP was performed which was unremarkable. During the ERCP, a sphincterotomy was performed with biliary sweep which was unremarkable with cytology showing benign reactive epithelial cells. Liver biopsy was also repeated which showed similar findings as prior. Throughout his course, the patient also did not have any fevers, right upper quadrant pain, or leukocytosis to suggest underlying infection including acute cholangitis. Bone marrow biopsy performed showed hypercellular (90%) with 3+ reticulin fibrosis and no increased blasts, consistent with underlying primary myelofibrosis. TB continued to rise to 29 mg/dL, and given negative extensive workup otherwise, the decision was made to start empiric treatment for possible PMF-related disease. Dynamic International Prognostic Scoring System (DIPSS) score was 2, correlating with intermediate risk. Ruxolitinib was initiated with significant improvement of TB to 18 mg/dL. 3 weeks after initiation, ruxolitinib had to be temporarily held due to adverse effects of bone marrow suppression, and TB subsequently increased to 35 mg/dL over the next 5 weeks. Ruxolitinib was then resumed at a lower dose with improvement again of TB to 17 mg/dL (Figure 4). The patient's course was unfortunately complicated by renal failure secondary to biopsy-confirmed bile-acid nephropathy, ascites, functional decline, and eventually was transitioned to hospice.

## 3. Discussion

Primary myelofibrosis (PMF) is a chronic myeloproliferative disorder with an incidence of about 1.5 per 100,000 per year, and predominantly occurs in middle-aged and older adults [1]. The pathophysiology of the disease involves the release of growth factors by megakaryocytes, with subsequent fibroblast hyperactivity and bone marrow fibrosis which lead to the characteristic systemic findings [2].

Liver involvement is common in primary myelofibrosis, with hepatomegaly reported in 40%–70% of patients, and nonspecific liver enzyme elevations are also commonly seen [3]. Mild elevations in serum bilirubin can be related to the ineffective erythropoiesis which occurs due to bone marrow fibrosis. This, in turn, can lead to intravascular hemolysis with a resultant mild indirect hyperbilirubinemia, elevated LDH, and low haptoglobin [4]. Our patient, in contrast, had a much more profound direct hyperbilirubinemia in addition to normal LDH and haptoglobin levels, which made an underlying hemolytic process unlikely.

In addition, extramedullary hematopoiesis (EMH) is a well-known phenomenon that occurs in PMF. EMH, a process in which hematopoietic tissue and blood cells develop outside of the blood marrow, develops as a compensatory response to the bone marrow fibrosis seen in PMF, and the liver and spleen are well-known sites of EMH [5]. Given its predilection for liver involvement, EMH has been suggested to be a rare etiology of obstructive jaundice. Progression of liver involvement can also lead to portal hypertension and associated complications such as ascites [6]. Although the ascites were not fully investigated further as the patient was transitioned to hospice care, we suspect that this may have been the etiology. However, when associated with biliary obstruction, EMH has typically been shown to have specific histologic findings on liver biopsy, in which hematopoietic precursors including megakaryocytes are diagnostic for EMH [6, 7]. As mentioned previously, both biopsies in our patient were nonspecific which can potentially indicate an inadequate sensitivity for liver biopsy findings. Given the temporal relationship between myelofibrosis treatment and TB improvement, we speculate EMH was likely the underlying etiology of his cholestatic liver injury.

In regards to lab abnormalities, PMF has also been associated with mild liver enzyme elevations, with ALP elevation being the most common abnormality. In the literature, cases with EMH-related ALP elevation have been associated with worse outcomes. Hyperbilirubinemia, however, is extremely uncommon in PMF, and the majority of patients have normal bilirubin levels [8]. Marked hyperbilirubinemia as a presenting sign of PMF progression has not been reported to date in the literature. We, therefore, suggest that even in patients with previously known and well-controlled PMF, a progression of disease should remain in the differential in cases of worsening hyperbilirubinemia.

Our case demonstrates that patients with PMF who present with hyperbilirubinemia can undergo extensive invasive workup before a diagnosis is made. Liver biopsy, although reported in the literature to be consistent with extramedullary hematopoiesis in such cases, can occasionally fail to show this as demonstrated in the 2 biopsies performed on our patient. In such cases, nonspecific findings of cholestatic liver injury can be suggestive of PMF-related liver injury. Furthermore, although ALP has been shown to be a prognostic marker, our case demonstrates that marked bilirubin elevations can also be associated with serious morbidity and even lead to complications including bile-acid nephropathy. When caring for patients with PMF that develop hyperbilirubinemia with unremarkable workup otherwise, we suggest a low threshold for empiric treatment of PMF, and as in our case, ruxolitinib can be extremely effective.
